# A Study of Neurological Soft Signs and Cognition in Schizophrenia

**DOI:** 10.7759/cureus.50925

**Published:** 2023-12-21

**Authors:** Yashika L Nathani, Abhijeet Faye, Vivek Kirpekar, Sushil Gawande, Rahul Tadke, Sudhir Bhave, Nishikant Ingole, Gulshan R Bandre

**Affiliations:** 1 Psychiatry, Gujarat Medical Education and Research Society Medical College, Vadodara, IND; 2 Psychiatry, Datta Meghe Medical College, Datta Meghe Institute of Higher Education and Research, Nagpur, IND; 3 Psychiatry, NKP Salve Institute of Medical Sciences and Research Centre and Lata Mangeshkar Hospital, Nagpur, IND; 4 Pharmacology, Jawaharlal Nehru Medical College, Datta Meghe Institute of Higher Education and Research, Wardha, IND; 5 Microbiology, Jawaharlal Nehru Medical College, Datta Meghe Institute of Higher Education and Research, Wardha, IND

**Keywords:** cognitive dysfunctions, negative symptoms, cognitive impairment, neurological soft signs, schizophrenia

## Abstract

Introduction: Neurological soft signs (NSS) are delicate neurological abnormalities that comprise deficits in motor coordination, problems with the sequencing of complex motor acts, and sensory integration difficulties. These are nonspecific with no specific localization in the brain. NSS are found in many patients with Schizophrenia. Cognitive dysfunctions are also present in more than two-thirds of patients with Schizophrenia. This study aims at assessing the NSS and its association with cognitive impairment in patients with Schizophrenia.

Methods: A total of 100 Schizophrenia patients were included in the study. The Heidelberg scale was used for assessing the NSS. The Montreal Cognitive Assessment Scale (MoCA) for cognitive impairment, the Positive and Negative Syndrome Scale (PANSS) for Schizophrenia, and the Brief Psychiatric Rating Scale (BPRS) were used to assess the severity. Statistical analysis was performed by Pearson’s Chi-square test, Kruskal-Wallis test, Wilcoxon rank tests and Spearman rank correlation along with mean and standard deviation.

Results: NSS were present in 68% (N=68) of the patients with motor coordination being maximally affected. Cognitive impairment was found in 73% (N=73) of patients with a MoCA score <26. Patients with predominant negative symptoms had higher NSS scores and lower MoCA scores. A “statistically significant” correlation was observed between cognitive impairment and NSS. Most patients with NSS and impaired cognition were in the "markedly ill" category of BPRS.

Conclusion: A significant association was observed between cognitive deficits, negative symptoms, and NSS in Schizophrenia. NSS and cognitive dysfunctions are integral parts of Schizophrenia symptom domains and need to be assessed as the negative symptoms and severity of illness are associated with NSS, especially problems with motor coordination and cognitive dysfunctions.

## Introduction

Schizophrenia is a progressive disorder affecting a person’s ability to think, feel, and act. Clinical features may consist of changes in emotions, thinking, cognition, perception, and behaviour. Neurological soft signs (NSS) are delicate neurological abnormalities like deficits in motor coordination, sensory integration, and the sequencing of complex motor acts [1]. These signs are nonspecific with no specific localization in the brain. The prevalence of NSS was found to be in the range of 50% to 65% [2,3], including chronic cases, first-episode patients and drug-naïve patients [1].

Cognitive impairment is commonly found in Schizophrenia and is a determinant of the treatment outcome of Schizophrenia. Cognitive symptoms include disorganized speech, thought disturbances, inattention, etc., which ultimately impair the individual’s ability to communicate. In addition to this, defects in working memory, speed of processing, and learning along with deficits in reasoning, planning, abstract thinking, and problem-solving have also been widely documented in Schizophrenia [4]. Almost 98% of patients diagnosed with Schizophrenia have these difficulties and they don’t reach their predicted cognitive functions later on [5].

In chronically ill Schizophrenia patients, NSS scores are significantly correlated with neuropsychological impairments [6].* *NSS has also shown a correlation with cognitive factors like memory (working and short term), executive functions and level of intelligence [7]. NSS have also been found significantly related to impairments in “psychomotor speed”/cognitive flexibility in chronic Schizophrenia [7]. Thus, there is significant evidence of NSS having an association with severe deficits in attention, executive functions, and memory.

Thus, neurocognitive tests and NSS can be considered different ways of assessing the same construct that is "brain functions". Having this background, this study was carried out with the aims of studying NSS and cognitive impairment and finding their association with each other and with various socio-demographic factors in patients with Schizophrenia.

## Materials and methods

Study design, setting and sampling

This was a cross-sectional study carried out in a tertiary health care facility for six months. After the Institutional Ethics Committee's permission, 100 Schizophrenia patients were admitted to a psychiatry ward and fulfilled the inclusion and exclusion criteria. Participants were selected by convenient sampling. As the prevalence of Schizophrenia is low, convenient sampling was done and a number of patients who visited the Psychiatry Department during the study duration and fulfilled the inclusion and exclusion criteria were included in the study. This led to a sample size of 100. Written informed consent was obtained either from the patient or a family member. The diagnosis of Schizophrenia was made using the “Diagnostic and Statistical Manual of Mental Disorders” (DSM-5) and through the clinical interview. Socio-demographic information was collected using the validated form prepared for the project.

Inclusion and exclusion criteria

Patients included in the study were those having Schizophrenia, between the age range of 18 and 60 years, admitted to a psychiatry unit and gave written informed consent. Patients reporting significant medical, surgical, or other psychiatric conditions making them unable to understand and answer the questionnaire, and those not willing to participate were excluded from the study. Each interview took around 30 minutes. The confidentiality of the participants was strictly maintained.

Tools for evaluation

The following scales were utilized for evaluation:

(1) Semi-structured Proforma

It consisted of a socio-demographic profile and clinical details (duration, type, severity, treatment, and course of Schizophrenia).

(2) “Brief Psychiatric Rating Scale” (BPRS)

This scale is used for assessing psychiatric symptoms and severity. It consists of 18 items. Severity is classified as mildly ill (score 18-32), moderately ill (33-44), markedly ill (45-55) and severely ill (>55). The total BPRS score has good reliability and validity [8].

(3) “Positive and Negative Syndrome Scale” (PANSS)

This is a 30-question scale consisting of seven questions for positive symptoms, seven suggestive of negative symptoms, and 16 symptoms of general psychopathology (GPS). It has good internal and test-retest reliability [9].

(4) “Montreal Cognitive Assessment Scale” (MoCA)

The MoCA scale assesses many cognitive functions like short-term memory, visuospatial abilities, executive functions, phonemic fluency, verbal abstraction, attention, concentration, and working memory. Language and orientation to time and place are also evaluated. Out of 30, a score of 26 or more is considered normal. The MoCA internal consistency coefficient (Cronbach’s alpha) is 0.867. The value of the intraclass correlation coefficient (ICC) value is 0.912 (P<0.01) [10].

(5) The Heidelberg Scale

This is a 16-element scale used for the assessment of negative syndrome scale (NSS). The minimum score is 0 and the maximum score is 48. The scale has five elements assessing “motor coordination”, three items assessing "integrative functions", two items assessing "complex motor tasks”, four elements assessing “right/left and spatial orientation", and two assessing "hard signs". It has good inter-rater, and test-retest reliability [11].

Statistical analysis

The scores obtained on different scales were summarized in terms of minimum and maximum, mean, SD (standard deviation), and CV (coefficient of variation). CV is the ratio of the SD to the mean. The lower the CV, the more the involvement of the component. The descriptive statistics for normalized NSS scores were obtained for each subscale and expressed in terms of CV. Comparison correlations were drawn using several statistical tests such as “Pearson’s Chi-square test”, “Kruskal-Wallis test”, “Wilcoxon rank tests”, and Spearman rank correlation along with mean and SD. Analysis was done with Statistical Package for the Social Sciences (IBM SPSS Statistics for Windows, IBM Corp., Version 20.0, Armonk, NY) and a p-value <0.05 was considered significant.

## Results

Sociodemographic and clinical profile

The majority (91%) of the participants were males and below 50 years of age. Forty-seven (47%) were educated up to the primary level, and most of the participants were unemployed. Fifty-six (56%) participants were unmarried, 30 (30%) were married, and the majority 58 (58%) were from rural areas. Thirty-nine (39%) had been experiencing Schizophrenia for 15 years or more. Seventy (70%) had a history of noncompliance with medication, and a family history of psychiatric illness was present in 39 (39%) participants (Table 1).

**Table 1:** Distribution of patients as per sociodemographic and clinical profile N: Number of participants

Characteristics	Levels	N (%)
Age (in years)	18-29	40 (40%)
	30-49	51 (51%)
	50-60	9 (9%)
Gender	Female	32 (32%)
	Male	68 (68%)
Marital status	Married	30 (30%)
	Unmarried	56 (56%)
	Widower/Widow	5 (5%)
	Separated/Divorced	9 (9%)
Education	Uneducated	10 (10%)
	Primary	47 (47%)
	High school	18 (18%)
	Higher secondary	13 (13%)
	Graduate and above	12 (12%)
Occupation	Unskilled	22 (22%)
	Semiskilled	15 (15%)
	Skilled	8 (8%)
	Unemployed	55 (55%)
Residence	Rural	58 (58%)
	Suburban	37 (37%)
	Urban	5 (5%)
Duration of illness (in years)	1-9	34 (34%)
	10-14	27 (27%)
	> = 15	39 (39%)
Adherence	Compliant	30 (30%)
	Non- Compliant	70 (70%)
History of deliberate self-harm attempt	Nil	79 (79%)
	Once	12 (12%)
	Multiple times	9 (9%)
Psychiatric treatment taken in past	Yes	92 (92%)
	No	8 (8%)
Family history of psychiatric illness	Positive	39 (39%)
	Negative	61 (61%)

The distribution of patients based on the severity of the disease on the BPRS showed that 46 (46%) participants had scores between 45 and 55, categorizing them as "markedly ill". This was followed by 42 (42%) patients with scores between 33 and 44, placing them in the "moderately ill" category. Additionally, 12 (12%) participants, with a score of >55, were classified as "severely ill" (Table 2).

**Table 2:** Distribution of patients as per the severity of Brief Psychiatric Rating Scale (BPRS) scores BPRS: Brief Psychiatric Rating Scale, N: Number of participants

BPRS Severity Grading	BPRS Severity	Frequency, N (%)
18-32	Mildly ill	0 (0%)
33-44	Moderately ill	42 (42%)
45-55	Markedly ill	46 (46%)
> 55	Severely ill	12 (12%)
Total		100 (100%)

According to the PANSS scale, 61 (61%) participants exhibited predominantly negative symptoms, while 39 (39%) predominantly displayed positive symptoms. The total PANSS mean ± standard deviation score was 97.81 ± 14.58.

Neurological soft signs

In this study, out of 100 participants, 68 (68%) had NSS on the Heidelberg scale. As shown in Table 3, among all scales, "motor co-ordination" showed the least CV (77.50%) and "hard signs" showed the highest CV of 100%. The maximum normalized mean score ± SD was found for the motor coordination subscale (0.40 ± 0.31). This states that, among the subscales of the Heidelberg scale, motor coordination was maximally affected and hard signs were minimally affected (Table 3).

**Table 3:** Descriptive statistics for raw and normalized scores of NSS according to the Heidelberg Scale CV: Coefficient of variation; NSS: Neurological soft signs

Heidel Berg Scale (NSS Present in 68%)	Scores		Normalization	CV
	Total	Mean ± SD	Mean ± SD	
Motor coordination	15	6.03 ± 4.61	0.40 ± 0.31	77.50
Sensory integration	9	1.75 ± 1.68	0.19 ± 0.18	94.74
Complex motor tasks	6	2.22 ± 1.77	0.37 ± 0.30	81.08
Spatial orientation	12	3.13 ± 2.87	0.26 ± 0.24	92.31
Hard signs	6	1.14 ± 1.15	0.19 ± 0.19	100.00
NSS - Total score	48	14.26 ± 10.01	0.30 ± 0.21	70.00

Cognition

In this study, 73 (73%) of the patients had scores <26, suggestive of impaired cognition. The minimum CV value obtained for the "orientation" subscale of MoCA was 17.98, followed by the "naming" subscale as 20.93. The total mean raw score of MoCA was 21.87 with an SD of ±4.50. These data show that orientation and naming were the most affected domains of cognition in MoCA (Table 4).

**Table 4:** Descriptive statistics for raw and normalized scores on MoCA MoCA: Montreal Cognitive Assessment; SD: Standard deviation; CV: Coefficient of variation

Scale	Raw Scores		Normalized	CV
	Total	Mean ± SD	Mean ± SD	
Visuospatial executive	5	2.97 ± 1.37	0.59 ± 0.27	45.76
Naming	3	2.58 ± 0.54	0.86 ± 0.18	20.93
Attention	6	4.40 ± 1.26	0.73 ± 0.21	28.77
Language	3	2.09 ± 0.75	0.70 ± 0.25	35.71
Abstraction	2	1.62 ± 0.60	0.81 ± 0.30	37.04
Delayed recall	5	2.88 ± 1.49	0.58 ± 0.30	51.72
Orientation	6	5.33 ± 0.94	0.89 ± 0.16	17.98
MoCA - Total score	30	21.87 ± 4.50	0.73 ± 0.15	20.55

A correlation between NSS and cognition (Heidelberg scale and MoCA) is observed (Table 5). The association between the two factors was obtained using the Chi-square test and it was found statistically significant (p< 0.01). The proportion of cases in the category of MoCA < 26 was significantly higher than in MoCA > 26, depicting the fact that NSS are more prevalent when cognitive impairment is high (MoCA<26). Also, the mean scores of NSS on each subscale were higher in patients with cognitive impairment (MoCA < 26) compared to those with normal cognition (MoCA ≥ 26).

**Table 5:** Correlation between NSS subscales and cognitive impairment (MoCA<26) *P-value: < 0.0001 (HS); Obtained using Pearson’s Chi-square test; HS: Highly significant; NSS: Neurological Soft Sign; MoCA: Montreal Cognitive Assessment

Heidel Berg Scale	MOCA Scale [Mean ± SD (Median)]		P-value*
	< 26 (n=73) NSS present - 61; NSS absent - 12	≥ 26 (n=27) NSS present - 7; NSS absent - 20	
Motor coordination	7.48 ± 4.02 (8.00)	2.11 ± 3.80 (0)	< 0.0001
Sensory integration	2.25 ± 1.63 (2.00)	0.41 ± 0.93 (0)	< 0.0001
Complex motor tasks	2.82 ± 1.56 (3.00)	0.59 ± 1.22 (0)	< 0.0001
Spatial orientation	3.99 ± 2.73 (4.00)	0.81 ± 1.78 (0)	< 0.0001
Hard signs	1.47 ± 1.12 (1.00)	0.26 ± 0.71 (0)	< 0.0001
NSS - Total	17.70 ± 8.56 (19.00)	4.96 ± 7.47 (1)	< 0.0001

When various subscales of the Heidelberg scale for NSS were correlated with different components of the MoCA scale, a significant negative correlation was obtained (p<0.05). Low scores on MoCA "subscales" were associated with higher scores on "each subscale" of NSS.

When the severity of Schizophrenia (on BPRS) was compared with the presence or absence of NSS, it was found that the majority of patients having NSS (52.94%) were in the "markedly ill" category, but the correlation was not statistically significant. Similarly, when the severity of Schizophrenia was compared with the normal or impaired score on MoCA, it was found that most patients with a MoCA score of <26 were in the "markedly ill" category, but this association was also not significant statistically. When the illness duration was correlated with cognition (MoCA), it was found that a significant proportion of participants who had been ill for more than 15 years had MoCA scores <26. (p<0.05). The correlation between NSS (present/absent) and illness duration was not significant.

The negative symptoms score of the PANSS scale also correlated significantly with impaired cognition (MoCA <26) with a p-value of <0.01 and with an NSS score (p<0.01). This indicated that the more negative symptoms, the higher the cognitive impairment and the greater the NSS (Table 6).

**Table 6:** Correlation between Cognition (MoCA) and NSS with PANSS MoCA: Montreal Cognitive Assessment; NSS: Neurological Soft Signs; PANSS: Positive and Negative Syndrome Scale

MOCA	PANSS, N (%)		Total	P-value
	Predominantly positive	Predominantly negative		
< 26	22 (30.14)	51 (69.86)	73	0.0058
≥ 26	17 (62.96)	10 (37.04)	27	
Total	39 (39)	61 (61)	100	
NSS	PANSS, N (%)			
Present	12 (17.65)	56 (82.35)	68	0.0001
Absent	27 (84.38)	5 (15.63)	32	
Total	39 (39)	61 (61)	100	

## Discussion

Cognitive impairment, negative symptoms, and neurological abnormalities such as soft signs influence the prognosis of Schizophrenia by having an effect on disease onset, treatment response, and subsequent functional recovery. Therefore, these are important aspects to be assessed for their presence and correlation. This study was an attempt to find these correlations. The mean age of the participants (in years) was 33.42, with a range of 18 to 60 years, which was similar to that described in the literature. According to a study done on Schizophrenia patients, most of the participants were in the age range of 20 to 39 years (69%) with a mean age (in years) of 32.02 [12].

In this study, the majority (68%) of the patients were males. This finding was corroborated with that of other studies [13,14]. The majority of patients were unmarried. Other studies also found the same [12,14]. This indicates that Schizophrenia usually begins in late teenage. Thirty-nine per cent of the patients had a duration of illness of more than 15 years. This result was corroborated by the findings of a study by Herold CJ et al. [15], where the number of patients with a disease duration of illness ≥15 years was found to be the highest.

Seventy per cent of the participants had a history of poor adherence to treatment. Previous data suggest that nearly 74% of patients discontinue medications due to various reasons such as poor insight, side effects and poor symptom control [16]. History of one or more deliberate self-harm attempts was present in 21% of the participants. It is known that suicide is a common reason for premature death in Schizophrenia. One study had shown a prevalence of deliberate self-harm attempts in patients with Schizophrenia comparable to the findings in the present study [17]. In this study, most of the other sociodemographic details, including the distribution of participants by education, occupation and residence, were similar to the findings of other well-known studies [13,14,18]. The authors specifically analyzed the correlation of education with cognition and NSS but found no significant association of education level with the presence and extent of cognitive dysfunction or NSS.

The high mean BPRS score of the participants reflects the fact that they had a moderate to severe level of behavioural disturbances (psychotic features) which required indoor psychiatric care. In the current study, the mean total score on PANSS was 97.81. The analysis of the PANSS scores of the participants suggested that the majority of the patients had predominant negative symptoms. A recent study reported the presence of at least one negative symptom in 57.6% of Schizophrenia patients, which is very similar to the percentage in our study [19]. Another study reported that 41% of the patients had >2 negative symptoms [20].

In this study, 68% of the patients had NSS. In a meta-analysis of NSS in Schizophrenia with a review of 33 studies, Chan RC et al. [21] found that the performance of an average of 73% of the participants on NSS was outside the range of non-Schizophrenic participants. In another NSS clinical study done on 100 patients with Schizophrenia, the authors found that the prevalence of NSS was 67%, which is in a similar range of prevalence in the current study [12].

Among all the subscales of the Heidelberg scale, motor coordination was maximally affected, followed by complex motor tasks. In a study conducted in Israel, the authors found similar results, that is Schizophrenia patients exhibited poor performance on the NSS in the motor coordination subscale [22]. In another study, participants scored the highest on the "motor coordination" component of the NSS scale, followed by "sensory integration" of the NSS [12].

In this study, 73% of the participants had impaired cognition. Schizophrenia is associated with abnormalities of cognition in many aspects like executive functions, attention, and memory that affect at least 80% of the patients and is clinically significant in predicting patient treatment and functional outcomes [23]. The mean score of MoCA was 21.87 in this study. In a study on cognitive testing in patients with Schizophrenia, the mean MoCA total score was found to be 22.75 which is almost similar to the finding in the index study.

The correlation of NSS with cognitive function showed a strong association between the two. The presence of NSS was positively correlated with cognitive impairment. An association was also found between different NSS and different domains of cognition in this study [24,25]. A specific correlation was also found between sensory and motor NSS and cognitive abilities that is spatial orientation, executive functions, and language performance [26].

Thus it can be said that a higher NSS score is related to the increased/severe deficits in any component of cognition on MoCA. In the present study, the authors observed that a lower score on the Visuospatial/Executive Function subscale (MoCA) was significantly associated with a higher score in complex motor tasks. Similarly, a higher score on any NSS subscale was associated with a lower score on the Attention subscale of MoCA. This observation was corroborated with that of other studies. In a study by Fawzi MM et al., in Psychiatric Outpatients Clinic, Egypt, in patients with Schizophrenia, it was found that patients with high NES (Neurological Evaluation Scale) scores had low Attention and Visuospatial or Constructional scores [27]. Chan RCK et al. [28], in their study using “Cambridge Neurological Inventory” (CNI) and “neurocognitive function tests” in Schizophrenia patients and controls, found moderate associations of NSS with impairment in executive function, attention, and verbal and visual memory. Frontal neurological signs have been shown to be correlated with visual-spatial memory, visuospatial processing and visuo-constructive tasks. Some other authors in their study found a significant association of "sequencing of complex motor acts" with executive function [29].

When correlations were drawn between the presence of NSS and the severity of the disease using BPRS, it showed that most of the participants were in the markedly ill category, indicating an association between NSS and severity of illness. As NSS are subtle neurological abnormalities that cause neurobehavioural problems and affect cognition, they can be considered as important predictors of the severity of the disease. Similar findings were noticed in other studies as well [30].

The severity of the illness was positively correlated with cognitive deficits. Cognition is an important symptom domain of Schizophrenia; hence, it is understood that an increase in severity is associated with poor performance in cognitive tasks including tests for attention, memory, executive functions, or visuospatial orientation. This finding was replicated in another study [31]. Longer duration of illness was associated with more cognitive impairment. This can be explained as Schizophrenia is a progressive disorder and cognitive impairment, along with other symptoms, worsens with time especially if not well treated. No correlation was found between education level and cognitive deficits.

The negative symptoms score in PANSS positively correlated with NSS, i.e., more the negative symptoms, the higher the NSS. In a study by Fawzi MM et al. [27], a significant positive association was found between high neurological signs and negative symptom scores compared to those having low neurological signs. Negative symptoms are associated with the neurological/brain insult, either neuro-developmental or degenerative, and NSS can also be traced to brain abnormalities. This explains the positive relationship of negative symptoms with NSS. NSS, cognitive impairment and negative symptoms are implicated consistently in structural abnormalities of brain areas (basal ganglia, cerebellum, somatomotor and somatosensory regions, areas involved in visual processing and spatial orientation) including dysfunction of frontoparietal networks. Thus, structural and functional biomarkers are common for NSS, cognition and negative symptoms explaining their association in Schizophrenia patients.

Cognition was also significantly altered in participants with higher negative symptom scores on PANSS. Srinivasan L et al. [32], in their study, observed that negative symptoms were correlated with cognitive dysfunction in all the domains including attention, executive functions and memory. Thus, cognitive dysfunction and NSS can be traced to a common etiology like neurological abnormalities and both these constructs are directly correlated with the severity of Schizophrenia.

Limitations

This was a cross-sectional, single-centre-based study with a smaller sample size. A large sample and multi-centred prospective studies may yield better results. Though this study helps in finding the association of NSS with Schizophrenia and cognitive dysfunctions, large-scale studies are needed for more valid results to generalize the type of association of NSS and cognitive dysfunction among patients with Schizophrenia. Ongoing medications/treatments (especially electroconvulsive therapies) may have an impact on cognition but this aspect was not analyzed in this study.

## Conclusions

This study highlights the statistically significant relationship between NSS and cognitive dysfunction, which also points to a possible neurological insult in Schizophrenia. NSS were found in the majority of the participants with motor coordination as the prominent sign. Cognitive dysfunctions were observed in more than one-third of the patients with Schizophrenia. Most of the patients having NSS and impaired cognition were in the markedly ill category. The negative symptoms of Schizophrenia had a positive correlation with NSS and cognitive impairment. Thus, NSS and cognitive dysfunctions are integral parts of Schizophrenia symptom domains and need to be assessed as the negative symptoms and severity of illness are associated with NSS, especially problems with motor coordination and cognitive dysfunctions.

Specific NSS, which are relatively easy to elicit than cognitive functioning, can be utilized to predict cognitive impairment in several domains in earlier stages of the disease. This, in turn, would help in early intervention. Thus, periodic monitoring of patients with Schizophrenia for NSS and cognitive impairment is essential and can be recommended for its early detection and management. This can help improve the treatment outcome and functioning of the treatment of patients.
